# System-wide analysis of the transcriptional network of human myelomonocytic leukemia cells predicts attractor structure and phorbol-ester-induced differentiation and dedifferentiation transitions

**DOI:** 10.1038/srep08283

**Published:** 2015-02-06

**Authors:** Katsumi Sakata, Hajime Ohyanagi, Shinji Sato, Hiroya Nobori, Akiko Hayashi, Hideshi Ishii, Carsten O. Daub, Jun Kawai, Harukazu Suzuki, Toshiyuki Saito

**Affiliations:** 1 National Institute of Radiological Sciences, 4-9-1 Anagawa, Inage-ku, Chiba-city, Chiba 263-8555, Japan; 2 Maebashi Institute of Technology, 460-1 Kamisadori-machi, Maebashi-city, Gunma 371-0816, Japan; 3 Mitsubishi Space Software Co., Ltd., Tsukuba-Mitsui Building, 1-6-1 Takezono, Tsukuba-city, Ibaraki 305-0032, Japan; 4 National Institute of Genetics, 1111 Yata, Mishima-city, Shizuoka 411-8540, Japan; 5 Maze Inc., 1-2-17 M604, Sennin-machi, Hachioji-city, Tokyo 193-0835, Japan; 6 Graduate School of Medicine, Osaka University, 2-2 Yamadaoka, Suita-city, Osaka 565-0871, Japan; 7 RIKEN Omics Science Center, 1-7-22 Suehiro-cho, Tsurumi-ku, Yokohama-city, Kanagawa, 230-0045, Japan; 8 RIKEN Center for Life Science Technologies, Division of Genomic Technologies, 1-7-22 Suehiro-cho, Tsurumi-ku, Yokohama-city, Kanagawa, 230-0045, Japan; 9 Department of Biosciences and Nutrition and Science for Life Laboratory, Karolinska Institutet, Stockholm, SE-141 86, Sweden; 10 RIKEN Preventive Medicine and Diagnosis Innovative Program, 2-1 Hirosawa, Wako, Saitama 351-0198, Japan

## Abstract

We present a system-wide transcriptional network structure that controls cell types in the context of expression pattern transitions that correspond to cell type transitions. Co-expression based analyses uncovered a system-wide, ladder-like transcription factor cluster structure composed of nearly 1,600 transcription factors in a human transcriptional network. Computer simulations based on a transcriptional regulatory model deduced from the system-wide, ladder-like transcription factor cluster structure reproduced expression pattern transitions when human THP-1 myelomonocytic leukaemia cells cease proliferation and differentiate under phorbol myristate acetate stimulation. The behaviour of MYC, a reprogramming Yamanaka factor that was suggested to be essential for induced pluripotent stem cells during dedifferentiation, could be interpreted based on the transcriptional regulation predicted by the system-wide, ladder-like transcription factor cluster structure. This study introduces a novel system-wide structure to transcriptional networks that provides new insights into network topology.

Transcriptional networks have been studied in relation to recurrent gene expressions patterns, which have been interpreted previously as cell types^1^. In the context of network structure, network motifs^2^ and a human transcriptional network among 119 transcription factors (TFs)^3^ have been reported. Hierarchical organization of modularity was described in *E. coli* metabolic networks^4^. Additionally, network dynamics have been examined based on relations between network motifs and dynamics^5^, and coordination of signalling and transcriptional responses have been observed^6^. Another approach, co-expression analysis, has been used to study functional gene modules^7^,^8^,^9^,^10^. Ruan *et al.* proposed gene modules related to a subtype of human lymphoma and to yeast telomere integrity based on co-expression analyses^7^. Remondini *et al.* reported a relationship between co-expression and the cascade of MYC-activated genes in rat^8^. Honkela *et al.* attempted to identify the targets of transcriptional factors (TFs) based on ordinary differential equation models^9^,^10^. However, so far, no system-wide structure involving the transition of expression patterns has been reported in transcriptional networks.

Here, we reveal a system-wide structure in a human transcriptional network based on co-expression analyses of temporal expression profiles. Briefly, our approach was: (i) eliminate irrelevant TFs by filtering TFs based on covariance of temporal expression profiles; (ii) identify interactions connecting the filtered TFs based on goodness-of-fit and slope ratio information using a co-expression model; (iii) divide the filtered TFs based on the goodness-of-fit to the co-expression model; (iv) infer a system-wide structure in the identified interactions based on statistical significance of the interactions between two classes; and (v) simulate expression pattern transitions based on a transcriptional regulatory model deduced from the system-wide structure. We applied a proven index^11^ to step (i) and a proven co-expression model^12^,^13^ to steps (ii) and (iii), to ensure that the approach was reliable and that the predicted structure was convincing. We deduced a system-wide, ladder-like transcription factor cluster structure and validated predicted recurrent pattern transitions by state transition simulations.

## Results

**Figure 1:**
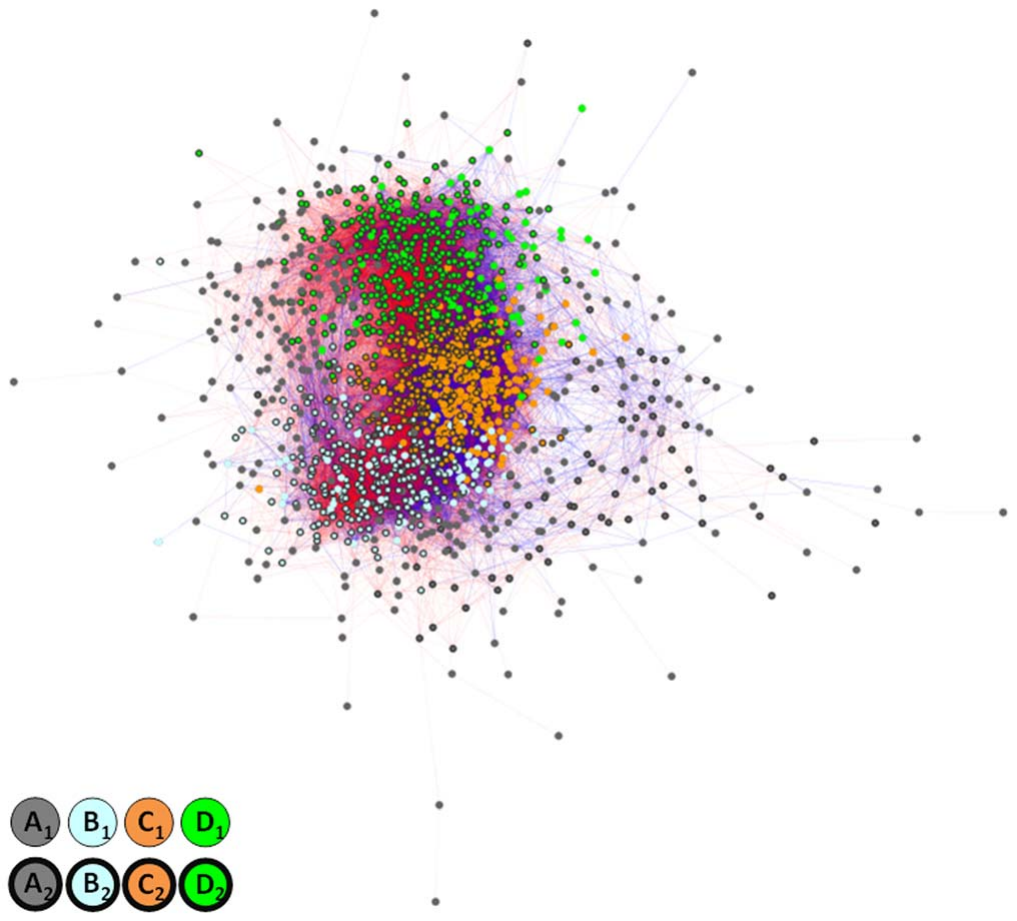
Transcriptional network of the filtered transcription factors. All the interactions satisfy two criteria, coefficient of determination *r*^2^ > 0.7 and the slope ratio <0.15. Red indicates promotive interaction; blue indicates inhibitory interaction. The network was drawn with Cytoscape^33^ using a force-directed layout. TFs are coloured according to their assigned classes (lower left corner).

We divided 2,247 TFs selected from the Genome Network Platform (http://genomenetwork.nig.ac.jp/index_e.html) into two groups, 1,619 TFs relevant to the transcriptional network and 628 TFs that were not relevant, based on the SUMCOV^11^ index in which covariance was calculated between temporal expression profiles of the TFs (see Methods, Supplementary Fig. S1 and TF_class_sumcov.xls at http://debe-db.nirs.go.jp/nw/ for details). Interactions connecting the filtered TFs were identified based on information provided by the co-expression model^13^ (see FltdTF.zip at http://debe-db.nirs.go.jp/nw/ for details). To identify interactions, we first selected the threshold of the goodness-of-fit to the co-expression model as 0.7, which retained almost all of the filtered TFs (99% = 1,606/1,619). Threshold values higher than 0.7 decreased considerably the number of TFs that remained (see Supplementary Fig. S2), even though the discarded TFs had been identified as relevant in the filtering stage. Next, we calculated the slope ratio (see Supplementary Fig. S3), and assigned a slope ratio threshold of 0.15, which is the same as the slope ratio threshold used in a previous study^13^. Consequently, 80,540 interactions that satisfied the goodness-of-fit (>0.7) and slope ratio (<0.15) criteria, were identified. These interactions connected 1,601 of the 1,619 relevant TFs (99% = 1,601/1,619) (Fig. 1).

**Figure 2:**
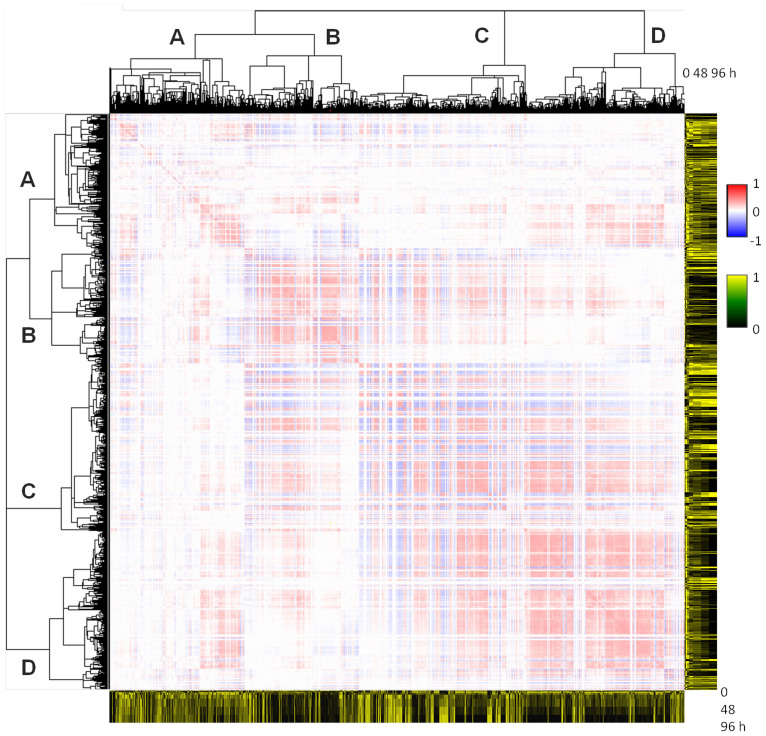
Goodness-of-fit matrices for the 1,619 filtered TFs in the transcriptional network. Each element (i, j) of a matrix indicates the goodness-of-fit, measured by the coefficient of determination *r*^2^, by the depth of the colour. Red indicates *b_ij_* > 0, where *b_ij_* is the slope coefficient^13^ between the temporal expression profiles of *i*-th and *j*-th TF, suggesting promotive regulation; blue indicates *b_ij_* < 0, suggesting inhibitory regulation. Letters on the dendrograms indicate the branch from which the TFs in the corresponding cluster split off. The heat-maps (right and below the matrices) show the temporal expression profiles of the TFs.

**Figure 3:**
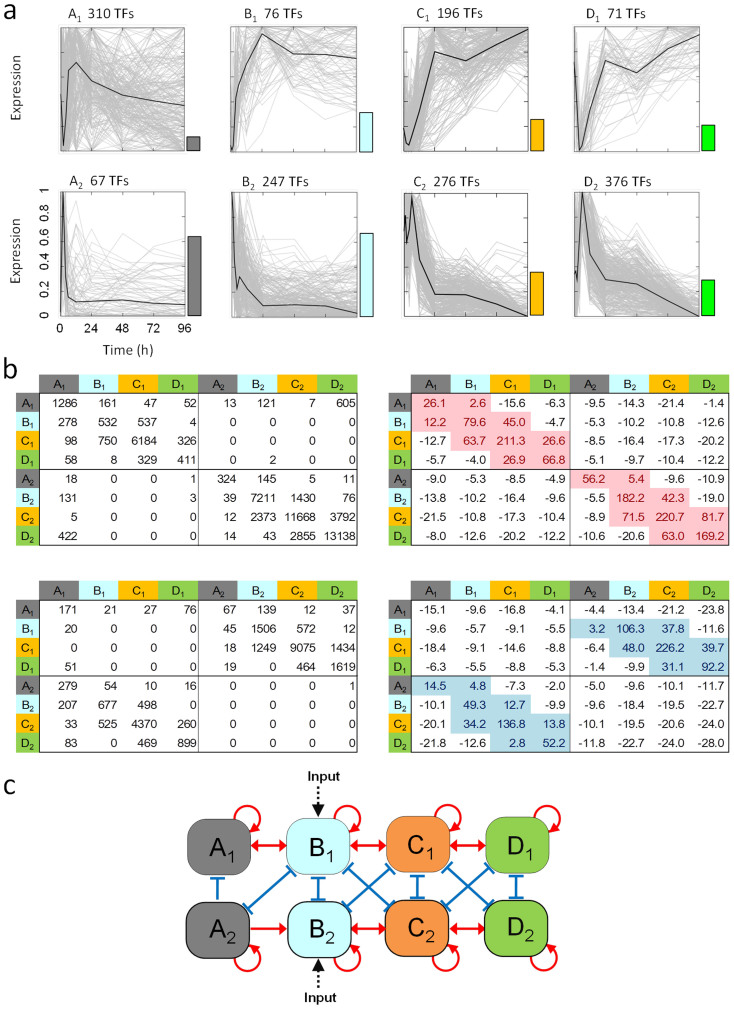
Identification of a system-wide transcriptional network structure. (a) Normalized temporal expression profiles of TFs in eight classes. Grey lines indicate the temporal profiles of the TF; black lines indicate the representative profile for each class defined as a series of medians. The length of the bar adjacent to each graph indicates the calculated similarity ratio between the unit step function and the representative profile (see Supplementary Fig. S4). The number of TFs assigned to each class is shown above each of the graphs. (b) Distributions of identified interactions between the eight TF classes. The two panels on the left show the numbers of identified interactions between the TFs in the corresponding classes; the upper and lower panel show the numbers of promotive and inhibitory interactions, respectively. The two panels on the right show the *Z*-scores based on the test statistic in the two-sample test for equality of proportions (see Methods for details); the upper and lower panels show the Z-scores for the promotive and inhibitory interactions, respectively. The *p*_0_ in the test statistic is the probability that an interaction with *r*^*2*^ > goodness-of-fit threshold and slope ratio < slope ratio threshold is observed by chance (the formula is given in Methods) and given as *p*_0_ = 2,180/628^2^ = 0.0055. The pink and blue shading indicates statistically significance as *p* < 0.005 (one-sided probability). The values (*i*-th row, *j*-th column) are for the interactions for which directionality^13^ was assigned such that the TFs in a class in the *i*-th row → the TFs in a class in the *j*-th column. (c) System-wide ladder-like structure of statistically significant inter-class interactions. External inputs were suggested to be supplied to classes B_1_ and B_2_.

To classify the TFs, we used an approach that differed from those used in previous studies^14^,^15^,^16^ where genes were grouped into clusters based on the expression profiles of the genes. In the present study, the genes were grouped into clusters based on the goodness-of-fit of the interaction; i.e., we grouped together two TFs that similarly interacted with third-party TFs (see Methods, and TF_class_sumcov.xls, FltdTF.zip and ClstView.zip at http://debe-db.nirs.go.jp/nw/ for details). As a result, four TF clusters were identified in the goodness-of-fit matrix (Fig. 2). The promotive (red) and inhibitory (blue) regulation patterns in the matrix for each cluster (Fig. 2) indicated that two types of TFs existed in each cluster, implying that further clustering was required. Therefore, we conducted a second clustering using a conventional clustering method, k-means clustering with k = 2, based on the temporal expression profiles. Four TF clusters composed of two types of classes were identified; one roughly showing an upward trend, A_1_, B_1_, C_1_ and D_1_, and the other showing a downward trend, A_2_, B_2_, C_2_ and D_2_ (Fig. 3a).

The identified classes were associated with interclass interactions as follows. The 80,540 interactions in the network were identified as promotive or inhibitory (see NM.cys at http://debe-db.nirs.go.jp/nw/ for details), and anchored to a combination of the two classes that included source TFs and sink TFs (Fig. 3b, left panels). A two-sample test^17^ for equality of proportions of the interactions identified 19 promotive and 17 inhibitory inter-class interactions as statistically significant (coloured elements in Fig. 3b, right panels). When the eight TF classes were connected with the statistically significant inter-class interactions, a system-wide structure that looked like two channels bridged by interfaces was revealed (Fig. 3c). We call this a system-wide, ladder-like transcription factor cluster structure. The TF-level view (Fig. 1) can be described as a force-directed-layout^18^ of the network, where the TFs are positioned by their connections based on mutual relationships. This view shows that the TFs in cluster B, C, and D are aggregated among themselves in the order B, C, and D, while the TFs in cluster A are positioned in peripheral parts of the network. The TF-level and class-level (Fig. 3c) views were consistent with each other.

The similarity of temporal profiles was evaluated between a representative profile of each class and a unit step function that modelled the external input by the phorbol myristate acetate (PMA) that was applied at the beginning of the experiments and supplied continuously over the entire experimental time course (see Methods and Supplementary Fig. S4 for details). The calculated similarities are indicated by the lengths of the vertical bars in Fig. 3a. The B_1_ and B_2_ classes in the two channels in the system-wide structure (Fig. 3c) both showed the highest similarities among the classes, and the further away a class was from B_1_ and B_2_, the lower the similarities of the class became. It is possible to speculate that the temporal profile of an external input will be deformed as the external input is processed in a channel. Based on this idea, the positioning of the classes (Fig. 3c) is reasonable because the similarity of a class with the step function decreased the further removed the class was from B_1_ and B_2_ (Fig. 3a).

**Figure 4:**
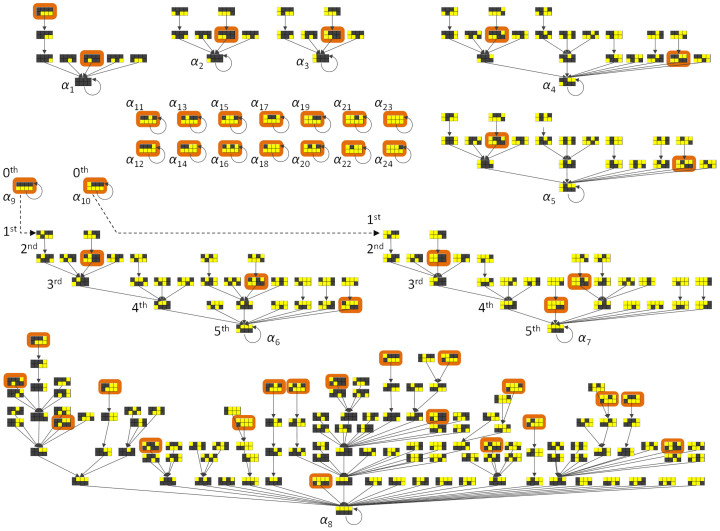
Computer simulations of state transitions in the system-wide, ladder-like transcription factor cluster structure. The states of the network structure are shown in the checkerboards, where the upper row, from left to right, indicates the state of the A_1_, B_1_, C_1_, and D_1_ classes, and the lower row indicates the state of the A_2_, B_2_, C_2_, and D_2_ classes. Solid arrows indicate state transitions where no step functions (described below) were supplied. Dashed arrows indicate state transitions where step functions (0 → 1 to class B_1_ and 1 → 0 to class B2) were supplied to mimic THP-1 cell differentiation under PMA stimulation. The ordinal numbers indicate state transitions after supplying the step functions, which model the external PMA input. Orange checkerboards indicate a final state when the enforced expression was added to the class B_2_ containing the MYC reprogramming factor under the condition without supplying the step functions which model the external PMA input. *Α*_i_ indicates an attractor of basin.

Computer simulations based on Boolean functions^1^ were performed to validate to proposal that the states transitioned and recurred to attractors towards which patterns of gene activity converged (Fig. 4). To simulate the PMA treatment, we supplied step functions, 0 → 1 to class B_1_ and 1 → 0 to class B_2_, as external inputs. The simulation results revealed eight basins of attraction that contained multiple states (for which the attracters were indicated as *α*_1_ to *α*_8_ in Fig. 4) and 16 singleton basins of attraction that contained one state (indicated as *α*_9_ to *α*_24_ in Fig. 4). Together, the 24 basins demonstrate multistability of the system-wide, ladder-like transcription factor cluster structure. Some of attractors showed expression patterns that were reminiscent of some cell types. Attractor *α*_1_, with an “ALL OFF” state, might suggest cell death (Fig. 4, upper left). Attractors *α*_6_, *α*_7_, and *α*_8_ (Fig. 4, bottom) mimicked the final expression pattern, determined by quantitative real-time reverse-transcription polymerase chain reaction (qRT-PCR) during human THP-1 cell differentiation under PMA stimulation^19^, in which the representative profiles of B_1_, C_1_, and D_1_ were upregulated and the representative profiles of B_2_, C_2_, and D_2_ were downregulated compared with their initial expression levels (Fig. 3a). Attractors *α*_9_ and *α*_10_ (Fig. 4, middle left) mimicked the initial expression patterns in which the representative profiles of B_1_, C_1_, and D_1_ were low while the representative profiles of B_2_, C_2_, and D_2_ were high compared with their final expression levels (Fig. 3a). When the external inputs were supplied, the trajectories moved out of attractors, *α*_9_ and *α*_10_, and into the basin of attractors, *α*_6_ and *α*_7_. After a trajectory moved into the basin of the *α*_6_ and *α*_7_ attractors, it converged there and did not return, even when the external inputs were de-actuated. This result demonstrated the irreversibility of the expression patterns, which is similar to the irreversibility of cell types after stem cell differentiation^20^. These results suggested that the trajectories mimic expression pattern transitions that occur when human THP-1 myelomonocytic leukaemia cells cease proliferation, become adherent, and differentiate into mature monocyte- and macrophage-like phenotypes under PMA stimulation.

The behaviour of a Yamanaka factor essential to dedifferentiate committed adult cells into a stem cell-like state^21^, MYC, was interpreted based on the system-wide, ladder-like transcription factor cluster structure. Our clustering approach positioned the stem cell reprogramming TF into the system-wide, ladder-like transcription factor cluster structure. MYC was positioned in class B_2_ as a hub node having promotive interactions with 34 downstream nodes (TFs) positioned in class B_2_ (see TF_class_sumcov.xls and NM.cys at http://debe-db.nirs.go.jp/nw/ for details). The simulation results showed that enforced expression of class B_2_ containing MYC maintained the state at a high level either in a basin or in a singleton basin (Fig. 4). These simulation results suggested that MYC activate the TFs that are closely associated, thereby maintaining the energy potential at a high level and keeping it in an undifferentiated proliferative state. MYC is notable among the Yamanaka factors^21^, as demonstrated in the effective generation of the induced pluripotent stem cells (iPSCs) through the control of histone acetylation^22^.

**Figure 5:**
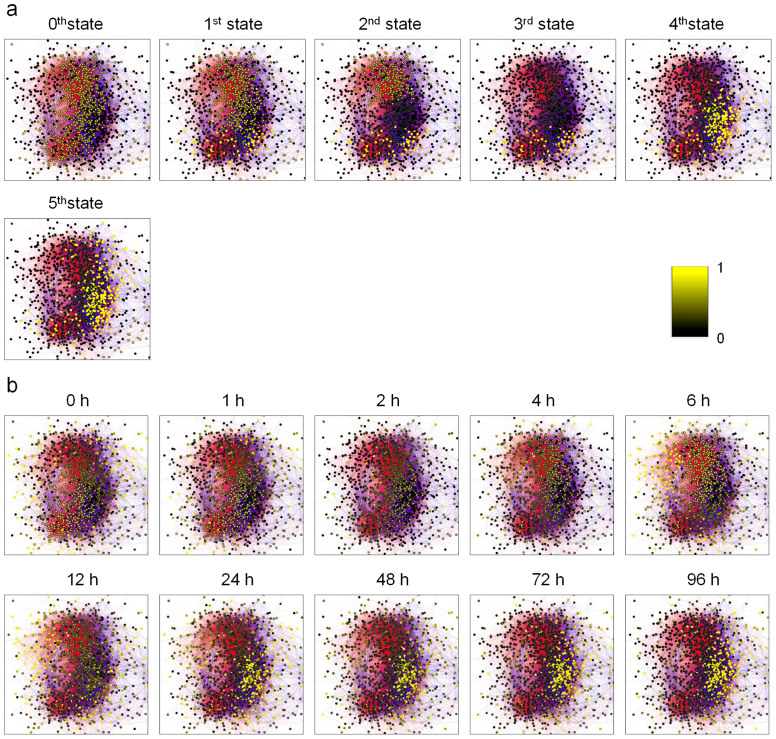
TF-level views of the simulated and actual expression pattern transitions in the transcriptional network. (a) Simulated expression patterns of the 0^th^ to 5^th^ states after supplying the step functions, 0 → 1 to class B_1_ and 1 → 0 to class B_2_, which modelled the external input by PMA. In these images the averaged expression levels between the two trajectories (from *α*_9_ to *α*_6_ and from *α*_10_ to *α*_7_ (Fig. 4)) are displayed. (b) Actual expression patterns 0 to 96 hours after starting PMA treatment. The coloured bar on the right hand side indicates the expression level of the TFs in both panels.

Our simulations were performed on a class level so that identical expression levels were assigned for TFs in a class. TF-level views of the expression patterns also showed that the simulation results mimicked actual expression patterns well (Fig. 5). The 0^th^ and 1^st^ states in the simulation results (Fig. 5a) mimicked high level expression over the network in the actual expression patterns from 0 h to 6 h after starting PMA treatment (Fig. 5b). The 2^nd^ to 5^th^ states in the simulation results (Fig. 5a) mimicked the spreading and aggregating areas of high level expression in the right-hand side of the network in the actual expression patterns from 12 h to 96 h (Fig. 5b). These observations further validated our system-wide network model.

## Discussion

Representative structure models for transcriptional networks have been reported previously; for example, (i) a recurrent network in which multiple TFs mutually coordinated their activity changes^19^; (ii) master regulator positioning at the top of fixed regulatory hierarchies^23^; (iii) a combination of models (i) and (ii) with switching from model (i) to (ii) as time passed^24^; and (iv) a three-layered model in which each layer included multiple TFs and intra-layer connections^3^. The system-wide, ladder-like transcription factor cluster structure revealed in the present study, is novel in that two module series were found to interact competitively.

The simulations based on the transcriptional regulatory model deduced from the system-wide, ladder-like transcription factor cluster structure indicated the validity of the network structure. The simulations successfully mimicked expression pattern transitions when human THP-1 myelomonocytic leukaemia cells ceased proliferation and differentiated under PMA stimulation (Fig. 4). Further, based on the transcriptional regulatory model from the system-wide, ladder-like transcription factor cluster structure, the behaviour of MYC during dedifferentiation could be interpreted, indicating that the factor may be essential for iPSCs. Importantly, the system-wide structure on which the transcriptional regulatory model was based is a novel concept (Fig. 3c).

A two phase model has been reported for the reprogramming process that induces iPSCs^24^. In this model, an early probabilistic phase of gene activation was followed by a later deterministic phase. Our simulation results can be interpreted equivalently; i.e., the diverse expression patterns in the early phase eventually converged into a fixed pattern of an attractor that recurred (Fig. 4). The simulations also suggested validity of the system-wide, ladder-like transcription factor cluster structure for the reprogramming process, because the results (Fig. 4) matched the interpretation based on the two phase model.

Additionally, the system-wide, ladder-like transcription factor cluster structure exhibited some of the properties of a self-organizing system^25^, namely, positive feedback and multiple interactions as well as a pattern at the global level that arose from numerous interactions among lower-level components (Fig. 1, 3c). The reproduction of the expression patterns in the transcriptional network could be interpreted based on the paradigm of self-organization; i.e., positive feedback by mutual inhibitions between two channels in the system-wide, ladder-like transcription factor cluster structure (Fig. 3c) autocatalytically amplified fluctuations of TF expressions (Fig. 3a), and external input by the induction of MYC triggered and maintained the process. As a result, the transcriptional network was pushed farther from equilibrium and reached high potential states that were maintained by the enforced expression (orange checkerboards in Fig. 4).

Furthermore, the system-wide structure described here can be linked with complex networks. The scale-free topology criterion can be defined as the coefficient of determination for a power-law distribution ~*ck*^−*γ*^, where *k* is the number of interactions per node and *γ* is the degree of decay^26^. For the predicted network (Fig. 1), the criterion was calculated as 0.67 (see Supplementary Fig. S5), which indicated that the predicted network had a near scale-free property. *Γ* was calculated to be 1.44 in the predicted network, similar to the value obtained in previous study based on co-expression analyses (*γ* = 1.19 in human^27^). This similarity further confirmed our inference of the system-wide, ladder-like transcription factor cluster structure, despite the structure being unique. Most importantly, the above observations imply that the approach described in this study uncovered an ordered structure that differed significantly from the homogeneous appearance (Fig. 3c) in the nearly scale-free network (Fig. 1), which had promised a homogeneous appearance.

## Methods

### Expression analysis

The expression profiles of 2,315 human TFs measured in a genome-wide dynamics analysis of a THP-1 cell line over a time course of growth, arrest, and differentiation were collected from the Genome Network Platform (http://genomenetwork.nig.ac.jp/index_e.html). qRT-PCR was performed using primer pairs generated automatically from a single exon of each target gene. To obtain high quality expression data, the reliability and specificity of each primer pair were confirmed in a preparatory experiment. Temporal expression profiles were observed at 0, 1, 2, 4, 6, 12, 24, 48, 72, and 96 hours after starting PMA treatment of human THP-1 cell during differentiation across two biological replicates. Human THP-1 myelomonocytic leukaemia cells cease proliferation, become adherent, and differentiate into a mature monocyte-and macrophage-like phenotype by stimulation with PMA^28^,^29^. By eliminating expression data with suspected measurement errors, 2,247 TFs were selected from the 2,315 available TFs. Of the 2,247 TFs, 1,350 (60%) were common to another independently developed dataset of 1,962 human TFs^30^. The averaged expression levels (copy numbers) of the two biological replicates were used for further analyses.

### Filtering TFs

The SUMCOV^11^ (sum of covariance) criterion was used to eliminate single TFs that were irrelevant to the transcriptional network. This criterion is based on the covariance matrix of the gene expressions assuming that there are groups of genes that are correlated among themselves, while being uncorrelated with the other groups. The SUMCOV for each TF was calculated using the averaged expression levels to identify the relevant genes. We implemented k-means clustering with k = 2 based on the logarithm of the SUMCOV provided by Gene Cluster 3.0^31^, to form a relevant and irrelevant group of genes. The cluster that included the TFs with the highest average criterion value was taken to represent the relevant group of genes for the network. The cluster that included the remaining TFs (representing the irrelevant group of genes) was used in a null-hypothesis to test significant interaction between the classes (see the “Inferring system-wide network structure” section in Methods for details).

### Identification of interaction

The temporal profiles were fitted to a linear co-expression model^13^, *x_j_* = *a_ij_* + *b_ij_x_i_*, that represented an interaction between the *i*-th and *j*-th TFs, where *x_i_* indicates the expression level of *i*-th TF. The coefficient of determination (*r*^2^) was calculated to determine the goodness-of-fit of the equation. The nodes connected by interactions with *r*^2^ values higher than the goodness-of-fit thresholds were investigated. We selected a goodness-of-fit threshold that left the highest numbers of filtered TFs in the network. Directionality of an interaction was assigned so that a small expression change in the ‘source’ gene was associated with a large change in the ‘sink’ gene. We chose a slope ratio threshold that imposed directionality on the interaction so that the ratio between the magnitudes of the slope coefficients (smallest/largest) in the linear model^13^ was less than the threshold.

### Clustering TFs

The filtered TFs were divided by the co-expression model into clusters based on a series of *r_ij_*^2^ values, *r_i1_*^2^, …, *r_ij_*^2^, …, *r_in_*^2^, where *r_ij_*^2^ indicates the *r*^2^ of a model in which the *i*-th TF regulates the *j*-th TF. Based on this method, if two TFs regulate all other TFs identically, the distance between the two *r_ij_*^2^ series will be zero. It is important to note that *r*^2^ is an absolute value that allows two interactions to be determined as identical based on their goodness-of-fit to the co-expression model, even if the effect of the interaction indicated by the sign of the slope coefficient *b_ij_* is different. Situations similar to this can be seen in the checkered patterns in Fig. 2.

Hierarchical clustering was performed using Gene Cluster 3.0^31^ with the Euclidean distance between the *r_ij_*^2^ series and the centroid linkage method. Clusters were visualized using Java Treeview^32^. The TF clusters were divided into two classes based on the temporal profiles of the TFs assigned to the clusters. For this clustering, we implemented k-means clustering with k = 2 of the normalized expression levels (between zero and one) with the Euclidean distance^31^. Suffix ‘1’ was assigned to the classes where the representative profiles showed an upward trend and suffix ‘2’ to was assigned to the classes where the profiles showed a downward trend.

### Inferring system-wide network structure

The effect of an interaction was decided based on the sign of the slope coefficient (*b_ij_*), which was estimated based on the co-expression model: promotive if *b_ij_* >0 and inhibitory if *b_ij_* <0. A two-sample test for equality of proportions was used to evaluate the statistical significance of the proportion (*p*_1_) of the interactions in the total number of potential *n*_i_ × *n*_j_ interactions between the classes, where *n*_i_ and *n*_j_ indicate the number of TFs contained in the classes. The null hypothesis was that *p*_1_ = *p*_0_, where *p*_0_ is the probability that an interaction, with *r*^2^ > goodness-of-fit threshold and slope ratio < slope ratio threshold, is observed by chance. The *p*_0_ was calculated based on the potential interactions between the TFs identified as single TFs (see OtherTF.zip at http://debe-db.nirs.go.jp/nw/ for details) that were irrelevant to the transcriptional network in the filtering analysis as (number of interactions, with *r*^2^ > goodness-of-fit threshold and slope ratio < slope ratio threshold, between single TFs)/(number of single TFs)^2^. The test statistic for the two-sample test for equality of proportions^17^ was: 
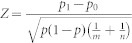
, where 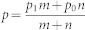, and *m* and *n* are the number of potential interactions between TFs in the corresponding two classes, and between TFs identified as single TFs, respectively. Additionally, we investigated the similarity between the representative profile of each class and the step function that modelled external input by PMA applied at the beginning of the experiments and supplied continuously over the entire time course (see Supplementary Fig. S4).

### State transition simulation

The network state transition was simulated based on Boolean functions (modified from Kauffman^1^). In the simulation, the states were presented as binary values (zero or one) at the class level. The significant interclass interactions were used to model transcriptional regulations in the network structure (see Supplementary Fig. S6). In the simulation of THP-1 cell differentiation under PMA stimulation, step functions (0 → 1 and 1 → 0) were provided to the B_1_ and B_2_ classes, respectively, as external inputs modelling the PMA stimulation.

## Author Contributions

K.S. conceived the study. C.O.D., J.K. and H.S. collected the data. K.S., H.O., S.S., A.H. and H.I. conducted the analyses. K.S. and H.N. developed the prediction method. K.S. and T.S. wrote the paper, and T.S. supervised the study. All authors discussed the results and commented on the manuscript. Note: RIKEN Omics Science Center ceased to exist as of April 1st, 2013, due to RIKEN reorganization.

## Supplementary Material

Supplementary Information Supplementary information
