# Supplementary material for: System-wide analysis of the transcriptional network of human myelomonocytic leukemia cells predicts attractor structure and phorbol-ester-induced differentiation and dedifferentiation transitions
Source: Sci Rep. 2015 Feb 6;5:8283. doi: 10.1038/srep08283 (PMC4319166; doi:10.1038/srep08283)
Supplement: Supplementary Information [file srep08283-s1.pdf]

Supplementary information

System-wide analysis of the transcriptional network of human myelomonocytic leukemia cells predicts attractor structure and phorbol-ester-induced differentiation and dedifferentiation transitions

Katsumi Sakata[1,2,*], Hajime Ohyanagi[1,2,3,4], Shinji Sato[5], Hiroya Nobori[3], Akiko Hayashi[1], Hideshi Ishii[1,6], Carsten O. Daub[7,8,9], Jun Kawai[7,10], Harukazu Suzuki[7,8], Toshiyuki Saito[1]

[1] National Institute of Radiological Sciences, 4-9-1 Anagawa, Inage-ku, Chiba-city, Chiba 263-8555, Japan
[2] Maebashi Institute of Technology, 460-1 Kamisadori-machi, Maebashi-city, Gunma 371-0816, Japan
[3] Mitsubishi Space Software Co., Ltd., Tsukuba-Mitsui Building, 1-6-1 Takezono, Tsukuba-city, Ibaraki 305-0032, Japan
[4] National Institute of Genetics, 1111 Yata, Mishima-city, Shizuoka 411-8540, Japan
[5] Maze Inc., 1-2-17 M604, Sennin-machi, Hachioji-city, Tokyo 193-0835, Japan
[6] Graduate School of Medicine, Osaka University, 2-2 Yamadaoka, Suita-city, Osaka 565-0871, Japan
[7] RIKEN Omics Science Center, 1-7-22 Suehiro-cho, Tsurumi-ku, Yokohama-city, Kanagawa, 230-0045, Japan[†]
[8] RIKEN Center for Life Science Technologies, Division of Genomic Technologies, 1-7-22 Suehiro-cho, Tsurumi-ku, Yokohama-city, Kanagawa, 230-0045, Japan
[9] Department of Biosciences and Nutrition and Science for Life Laboratory, Karolinska Institutet, Stockholm, SE-141 86, Sweden
[10] RIKEN Preventive Medicine and Diagnosis Innovative Program, 2-1 Hirosawa, Wako, Saitama 351-0198, Japan

[*] Corresponding author: ksakata@maebashi-it.ac.jp

[†]RIKEN Omics Science Center ceased to exist as of April 1st, 2013, due to RIKEN reorganization.

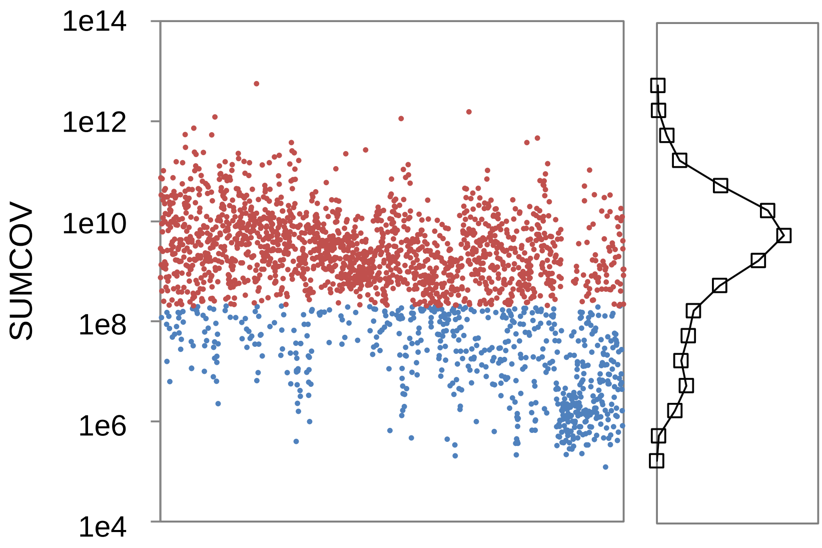

Figure S1. Distribution of the SUMCOV indices for the 2,247 TFs. Red dots indicate TFs classified as ‘relevant’; blue dots indicate TFs classified as ‘irrelevant’. The diagram on the right shows the frequency of the TFs.

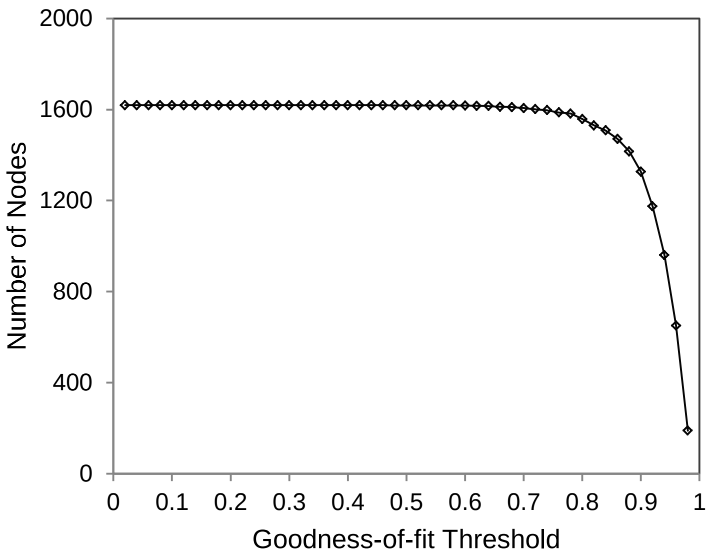

Figure S2. Number of nodes connected by the interactions with r^2 (coefficient of determination) higher than the goodness-of-fit threshold.

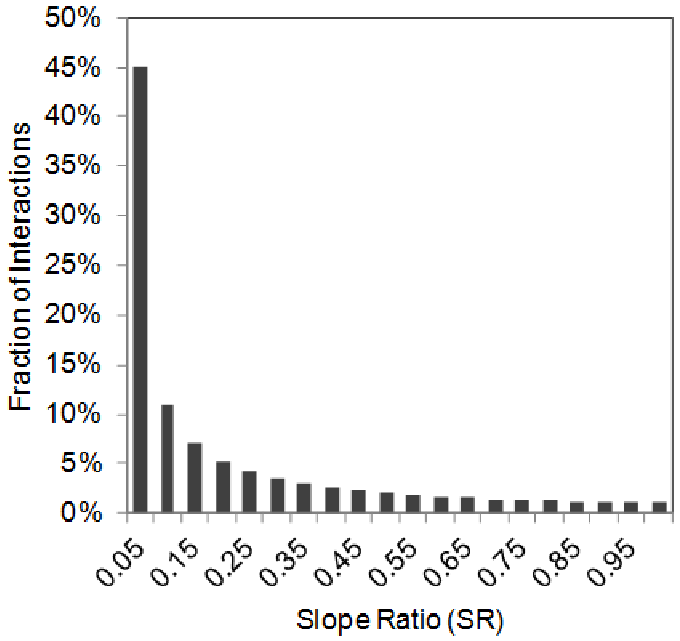

Figure S3. Distribution of the slope ratio values for the 127,599 interactions obtained at the goodness-of-fit threshold of 0.7.

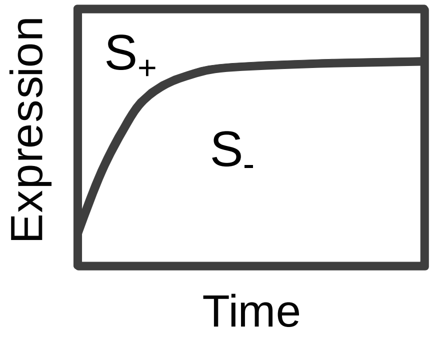

Figure S4. Temporal profile evaluated between a representative profile of a TF class and a unit step function that modelled the external input by phorbol myristate acetate (PMA). The ratio that indicates similarity between a unit step-function and a representative profile was calculated as the maximum between S_+/S_- and S_-/S_+, where S_+ indicates the area over a representative profile and S_- indicates the area under the profile.

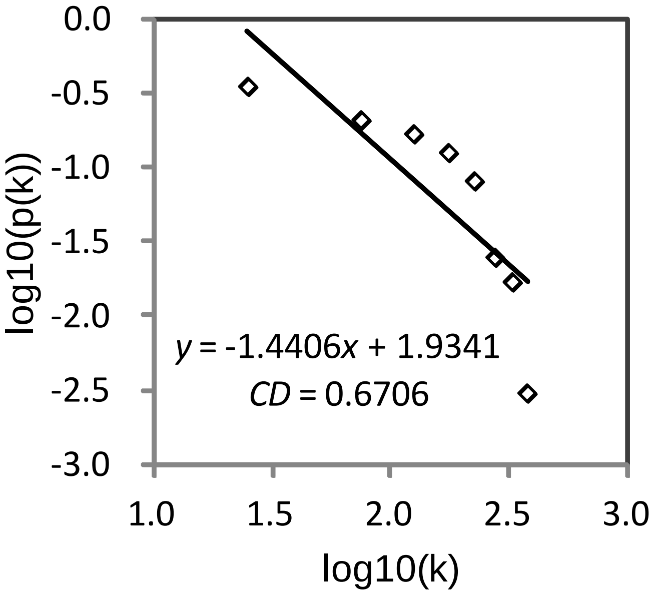

Figure S5. Determination of the scale-free topology criterion for the predicted network (Fig. 1). The x-axis shows the logarithm of the number of nodes connected by an edge and the y-axis shows the logarithm of the ratio of these nodes over all the nodes. The equation is the linear approximate equation between the x- and y-axes. *CD* is the coefficient of determination for the equation. The degree of decay is estimated as minus the slope of the line in the figure. Each edge represented in the figure was analysed as an undirected edge.

a

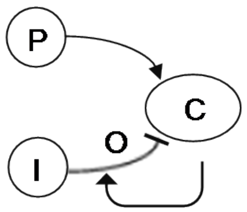

b

Class	Inhibitory input	Operator
0	0	0
0	1	1
1	0	0
1	1	0

Promotive input	Operator	Class
0	0	0
0	1	0
1	0	1
1	1	0

Figure S6. State transition simulation based on Boolean functions. (a) Schematic representation of the Boolean functions used in the simulation. P is the promotive input, I is the inhibitory input, O is the operator, and C is the TF class. The directed edge from P is indicated by red arrows in Fig. 3c. The directed edge from I is indicated by a blue T-bar in Fig. 3c. This edge blocks activation of the class unless it is itself inhibited by the activation of the class. (b) Effect of the “NOT IF” Boolean function. The table (left) shows the “NOT IF” Boolean function describing regulation of an operator by a class state and inhibitory input. For an operator (O), 1 = enabled and 0 = disabled. For a class (C), 1 = active and 0 = inactive. The table (right) shows the “NOT IF” Boolean function describing activation of a class. Activation requires that the promotive input (I) is active (1), and the operator is disabled (0).In Fig. 3c, multiple T-bars to a class are considered as at least one input (1) that guarantees inhibition is enabled (1); multiple arrows to a class are considered as at least one input (1) that guarantees promotion is enabled (1) in the upper channel (A_1, B_1, C_1, and D_1); all inputs (1) are required to guarantee promotion is enabled (1) in the lower channel (A_2, B_2, C_2, and D_2).
